# Migraine and stress—an exploratory cross-country study of external stress factors

**DOI:** 10.1186/s13104-021-05587-8

**Published:** 2021-05-08

**Authors:** Irene van Staveren

**Affiliations:** Institute of Social Studies, Erasmus University Rotterdam, Kortenaerkade 12, 2518 AX The Hague, The Netherlands

**Keywords:** Stress, Migraine, Conflict, Work-stress, Civil war, Migrants, Productivity, Unemployment, Cross-country analysis

## Abstract

**Objective:**

The data collected by the Global Burden of Disease 2016 project indicate that migraine ranks second in high-income countries with very competitive and flexible labour markets, and first in low- and middle-income countries suffering from civic unrest and conflict. This raises the question whether external stress factors may be correlated with migraine years lived with disability per 100,000 inhabitants (YLD). The objective of this exploratory study is to test the hypothesis that external stress factors are correlated with the prevalence and severity of migraine at the country level. The analysis uses two country groups: developed and developing countries. For the first group, the proxy variables for stress are labour productivity and unemployment rate. For the second group, the proxy variables measure conflict-related deaths and share of migrant/refugee population.

**Results:**

The results show a positive relationship between the stress variables on the one hand and migraine YLD on the other hand for both country groups. Almost all results are statistically significant at *p* < 0.01. These exploratory findings suggest that societal stress factors may be potential candidates for modifiable factors for the prevalence and/or severity of migraine at the country level.

## Introduction

According to the Global Burden of Disease (GBD) 2016 project, migraine ranks first for the age group of 15 to 49 years while it ranks second for all ages, measured as Years Lived with Disability per 100,000 inhabitants (YLD) [1]. An insightful GBD study focusing on migraine reveals that there are 1.04 billion migraine patients worldwide who together suffer from 45.1 million years lived with disability [2]. Interestingly, migraine ranks high in all countries in the world and, contrary to beliefs, no significant relationship between the prevalence of migraine and socio-economic status has been found. The socio-economic development of a country “is not a major determinant of the size of the headache burden”, the authors conclude ([2], p. 971).

The causes of the debilitating chronic disease are largely unknown and there is no effective treatment yet. As a consequence, migraine patients often suffer for years if not decades, which explains the high ranking in terms of disability. Studies into possible causes, comorbidities, and treatments are largely limited to clinical studies (RCTs and cohort studies). There are no systematic cross-country analyses available with risk factors for migraine. Clinical studies suggest three categories of candidates for modifiable risk factors: metabolic factors, mental health factors, and hormone factors [3–8]. For all these risk factors, chronic stress is increasingly mentioned as a key mechanism in the development and chronification of migraine [9].

A few small cohort studies have shown statistically significant relationships between migraine and external stress factors, in particular work-related stress [10, 11] and stress from civil war, terrorism, and combat [12–14]. But this has not yet been analyzed at the cross-country level. Why is migraine the number one chronic disease among those of working age in developed countries? And why is migraine the number one chronic disease in countries such as Afghanistan, Syria, Venezuela, Colombia, Jordan, Iraq, Lebanon and Palestine? In this article, I will explore these questions statistically. The results should be taken with much caution—they are exploratory in character. The purpose of the present study is not to establish causality, but to complement findings from clinical research and inform research about modifiable risk factors for migraine.

## Main text

### Literature review

Migraine is a neurological disease with a cascade of effects in the brain, in which the HPA-axis (Hypothalamic–Pituitary–Adrenal axis) and the trigeminal nerve (with one of the branches behind the eyes, where migraine headache is often located) play a key role [15–18]. Genetic research shows more and more genes that are related to migraine, while the fact that some patients develop migraine late in their lives and others report a sudden end to the attacks suggests that epigenetic factors, possibly related to stress, may play a role too [19].

The HPA-axis appears to play a crucial role not only in stress but also in migraine, when the HPA-axis seems to be overactive and the body does not have sufficient time to recover and to bring cortisol, blood pressure, glucose, oestrogen, pulse, and breathing back to normal values [20–22]. Stress researchers refer to that overactivity as allostatic overload [22]. Neuroimages of individuals suffering from chronic stress show structural and functional changes in the amygdala, hippocampus and prefrontal cortex [22]. Over the past decade, allostatic overload has also been related to migraine, not only from migraine to stress, but more importantly, from stress to migraine, which suggests a possible causal path [7, 15, 23]. The brains of migraine patients appear to be highly sensitive, in particular the hypothalamus, amygdala, and prefrontal cortex, which show similar structural and functional changes as in the brains of those suffering from chronic stress—even outside attacks [18, 24, 25].

The modern flexible work-context as well as civic unrest and conflict are dominant external stressors for large populations. They may, therefore, have an aggregate effect on migraine YLD. A recent editorial in *Neurology* concludes that various external factors “can affect the threshold for migraines, including stressful life events ([26], p. 53).” Researchers have pointed in particular at work-related stress in the western world, related to an individualist, competitive work culture in ever more flexible and insecure labour markets, with continued outsourcing of low skilled work, job replacement by modern technology and 24/7 digital availability [27]. For the developing world, basic insecurity of life is likely to be a source of chronic stress, related to civil war, violent conflict, weak states, large-scale human rights abuses, and large refugee flows due to crises in neighbouring countries [26, 28].

### Methods

No cross-country studies on migraine and stress are available. Due to this gap, the aim of this study is to explore the extent to which stress might be a possible modifiable factor for migraine at the cross-country level. The setting is a cross-country analysis with secondary data on migraine and stress for two country groups (developed and developing countries), each with their own set of salient stress proxy variables, using the OLS regression method. Complete data was available for almost every country in the world: all developed countries (38 in total, all OECD members and affiliates) and 153 developing countries (almost all remaining countries). The cross-section method does not establish causality due to the lack of time variation. However, with migraine as the dependent variable, reverse causality is not likely at the country-level, contrary to a possible two-way relationship between migraine and stress at the individual level.

Migraine was measured as YLD per 100,000 inhabitants, for all ages and for the age group 15–49 years old (which is the group most affected by migraine), respectively. The independent variables were deliberately chosen to be exogenous stress factors. For the developing country group, two proxy variables for external stress were used, related to war, conflict and insecurity. The first is the presence of high-intensity battle-related deaths (with a minimum of at least 1000 deaths in a year) from data collected by the Uppsala Conflict Data Program [30]. In order to account for the low number of countries with battle-related deaths, I have transformed the data into a binary variable with 0 for deaths below 1000 and 1 for 1000 or more deaths per country. The second proxy variable is international migrants (including refugees) as share of the population in the receiving country [31]. For the group of developed countries, the independent variables are two proxy variables for work-related stress. The first is the unemployment rate, reflecting labour market insecurity, in particular in the context of labour market flexibilization. The second proxy variable is labour productivity, measured as output (GDP in US dollars) per worker and signalling work pressure [32]. Together, the two labour market variables represent the carrot and the stick of flexible labour markets.

Table 1 shows the descriptive statistics for both country groups. The summary makes clear that all variables used in the analysis are continuous variables (scale level of measurement) except for the new battle-related deaths variable (a binary variable) because data was available for only 40 countries, including cases with less than ten deaths and countries with military casualties abroad on UN peace keeping missions. Finally, all data concern the year 2016—the year to which the GBD data on migraine refer.

**Table 1:** Descriptive statistics for migraine and stress factors (2016)There is an error in the numbers in the table: first row Maximum reads "075" but should be "1075)"

Developed countries	Mean	Standard deviation	Minimum	Maximum	No of countries
Migraine all ages (YLD per 100,000 inhabitants)	778	140	479	1075	38
Migraine 15–49 years (YLD per 100,000 inhabitants)	1063	212	675	1519	38
Labour productivity (GDP per worker)	81,135	47,089	22,427	235,385	38
Unemployment rate (% unemployed in the labour force)	7.39	4.10	2.98	23.54	38

**Developing countries**					
Migraine all ages (YLD per 100,000 inhabitants)	623	139	301	1206	155
Migraine 15–49 years (YLD per 100,000 inhabitants)	881	170	475	1619	155
Battle Related Deaths (absolute number of deaths)	2157	7098	4	41,340	40
Battle Related Deaths > 1000 (ordinal variable: 0 for < 1000 and 1 for > 1000)	0.06	0.235	0	1	155
Migrants as share of population (%)	8.40	14.95	0.07	88.4	153

*YLD* Years Lived with Disability, *GDP* Gross Domestic Product in US dollars

### Results

Two similar models are estimated, one for developed and the other for developing countries:

Model for developed countries: ${\text{M = }}\alpha {\text{LP + }}\beta {\text{UR + }}\varepsilon.$

In the OLS-estimation, the dependent variable, M, is measured as Years Lived with Disability per 100,000 inhabitants (YLD). In addition to migraine for all ages, the estimation was repeated for migraine in the age group 15–49, which is the age group that suffers most from the disease, hence, this group has higher YLD-values. The independent variables are labour productivity (LP), which is measured as annual output (GDP) per worker, and unemployment rate (UR), α and β are the respective parameters, while ε refers to the error term. The analysis is cross-section for OECD countries plus affiliate countries for which OECD also collects data (n = 38).

Model for developing countries: ${\text{M = }}\alpha {\text{BRD + }}\beta {\text{MIG + }}\varepsilon.$

In the OLS-estimation, the dependent variable, M, is again measured as Years Lived with Disability per 100,000 inhabitants (YLD) for all ages and for the age group 15–49 years. The independent variables are Battle Related Deaths over 1000 (BRD), measured as a binary variable with the value of 1 for at least 1000 deaths per year and 0 otherwise, and the share of migrants in the population (MIG), and α and β are the respective parameters, while ε refers to the error term. The analysis is cross-section for all developing countries for which all data is available (n = 153).

For developed countries, Table 2 shows the results for migraine all ages and migraine in the 15–49 years age bracket—groups that are made for the sake of relevance since migraine is most prevalent in the specified age group (the purpose of having two groups is not a comparison between two independent groups, because the second group is part of the first group). All parameters are statistically significant at a 99% confidence interval (*p* < 0.01) but the conventional confidence level of 95% is reported in the table. All parameter signs are in the expected direction and the regression line has a constant, which is statistically significant. The results can be interpreted as follows: when labour productivity increases with 1000 US dollar per year, migraine increases with one YLD per 100,000 inhabitants for all ages (*p* = 0.001) and two YLD per 100,000 inhabitants for the age bracket of 15–49 years (*p* < 0.001). When the unemployment rate increases with 1 percentage point (for example from 7 to 8 percent), migraine increases with 19 YLD per 100,000 inhabitants for all ages (*p* < 0.001) and 29 YLD per 100,000 inhabitants for the age group of 15–49 years (*p* < 0.001).

**Table 2:** Results for migraine YLD per 100,000 inhabitants in developed countries (2016)

	Migraine all ages	*p*-value	CI (95%)	Migraine 15–49 years	*p*-value	CI (95%)
Labour productivity (GDP per worker)	0.001	0.001	0.001–0.002	0.002	< 0.001	0.001–0.003
Unemployment rate (% unemployed in the labour force)	19.235	< 0.001	10.688–27.782	28.841	< 0.001	16.739–40.942
Constant	527.889	< 0.001	429.840–625.937	652.628	< 0.001	513.806–791.450
R^2^	0.685			0.735		
N	38			38		
F-statistic	15.462	< 0.001		20.584	< 0.001	

*YLD*  Years Lived with Disability, *GDP* Gross Domestic Product, *CI* Confidence Interval. R^2^ provides the model’s goodness of fit (in percentages of total explanatory power) with the F-statistic as its level of significance. See Table 1 for mean and standard deviation of all variables

For developing countries, Table 3 shows the results. Again, the results are split out for migraine all ages and migraine in the 15–49 years age bracket – groups that are only made for the sake of relevance since migraine is most prevalent in the specified age group.

**Table 3:** Results for migraine YLD per 100,000 inhabitants in developing countries (2016)

	Migraine all ages	*p*-value	CI (95%)	Migraine 15–49 years	*p*-value	CI (95%)
BRD > 1000 (binary variable)	66.431	0.148	− 23.834–156.695	192.595	0.001	81.300–3-3.889
Migrants as share of population (%)	2.836	< 0.001	1.411–4.261	1.585	0.077	− 0.172–3.342
Constant	596.779	< 0.001	571.673–621.885	859.655	< 0.001	828.700–890.610
R^2^	0.100			0.085		
N	153			153		
F-statistic	8.350	< 0.001		6.949	< 0.001	

*YLD* Years Lived with Disability. BRD > 1000 = Battle Related Deaths (binary variable: 0 for < 1000 and 1 for > 1000). *CI* Confidence Interval. R^2^ provides the model’s goodness of fit (in percentages of total explanatory power) with the F-statistic as its level of significance. See Table 1 for mean and standard deviation of all variables

Two of the four parameters of the independent variables are statistically significant at a confidence interval of 99% (*p* < 0.01) and one parameter with a confidence interval of 90% (*p* < 0.1). All parameter signs are in the expected direction and the regression line has a constant, which is statistically significant. The interpretation of the results is as follows. When the number of battle-related deaths increases from less than 1000 to more than 1000, migraine increases, on average, by 66 YLD per 100,000 inhabitants for all ages (*p* = 0.148) and 192 YLD per 100,000 inhabitants for the age group of 15–49 years (*p* = 0.001). When the share of migrants in the population increases by 1 percentage point (for example from 2 to 3%), migraine increases by 2.8 YLD per 100,000 inhabitants for all ages (*p* < 0.001) and 1.6 YLD per 100,000 inhabitants for those between 15 and 49 years old (*p* = 0.077).

### Discussion

The results suggest that, while for developed countries work stress factors show to be associated with migraine, for developing countries civic unrest and conflict factors show to be associated with migraine (although the model for developing countries has a much lower goodness of fit). These results at the cross-country level support the findings from clinical studies. However, more detailed research is necessary to understand which modifiable risk factors would be the most salient in preventing and reducing migraine. Some migraine researchers suggest that migraineurs are particularly susceptible to environmental changes, which may lead “to inappropriate processing or interpretation of stressful information ([25], p. 593)” But we need to understand much better the mechanisms through which environmental stress (as compared to individual-level stress) would affect the prevalence and severity of migraine. RCT studies are not suited to unveil mechanisms, whereas regression analysis may help to identify possible causal factors. Some researchers have developed hypotheses about migraine as a maladaptive response to life in stressful environments, such as today’s globalized, flexible, and complex societies, but the mechanisms behind such a maladaptive stress response are yet under-researched [29].

It may, therefore, very well be that stress and migraine may mutually reinforce each other in a process involving stress factors at various levels – individual, local context-related, and country-level. This requires multi-level research, including at the cross-country level.

## Limitations

There are several limitations of this study. First, there is a lack of detailed stress-indicators at the country level. It would be helpful, for example, if there would be representative national survey data available on types of stress and the severity of stress experienced, disaggregated by age group. Second, the GBD data are the result of model estimations, which implies uncertainty in the reliability of the measurement of the dependent variable. Over time, migraine statistics are likely to be more precise but for now, only model estimations are available, which tend to have higher standard errors for developing countries. Third, the OLS regression relies on cross-sectional data and has no time-dimension. There exist GBD data of earlier years, but migraine has traditionally been underdiagnosed, in particular in developing countries, so using data from before 2016 is not advisable for improving the quality of the estimations. Future GBD estimations of migraine YLD may be used for panel data analysis, allowing for a time-dimension in the regression analysis.

## Data Availability

All data are available in the public domain, from GBD, OECD (based on ILO data), World Bank, and University of Uppsala. The dataset used and analysed during the current study is available from the author upon request.
